# Aging reimagined: Bridging clinical modulation and scientific breakthroughs

**DOI:** 10.5114/biolsport.2026.156224

**Published:** 2025-11-24

**Authors:** Salah Mhamdi, Karim Chamari, Ahmed S. BaHammam, Walid Ahmed Alkeridy, Abdulrahman Ahmed Aldeeri, Helmi Ben Saad

**Affiliations:** 1 University of Sousse, Faculty of Medicine ‘Ibn el Jazzar’ of Sousse, Sahloul University Hospital, Department of Anesthesia and Intensive Care, Sousse, Tunisia; 2 Anesthesia department, King Khalid Hospital, Najran, Kingdom of Saudi Arabia; 3 Naufar Center, Doha, Qatar; 4 Higher Institute of Sport and Physical Education of Ksar Said, University of Manouba, Manouba, Tunisia; 5 University Sleep Disorders Center, College of Medicine, King Saud University, Riyadh, Saudi Arabia; 6 King Saud University Medical City (KSUMC), King Saud University, Riyadh, Saudi Arabia; 7 Department of Medicine, College of Medicine, King Saud University, Riyadh, Saudi Arabia; 8 Department of Medicine, Division of Geriatric Medicine, University of British Columbia, Vancouver, BC, Canada; 9 General Administration of Home Health Care, Therapeutic Affairs Deputyship, Riyadh, Saudi Arabia; 10 University of Sousse, Faculty of Medicine ‘Ibn el Jazzar’ of Sousse, Farhat HACHED University Hospital, Research Laboratory LR12SP09 ‘Heart Failure’ Sousse, Tunisia; 11 Department of Physiology and Functional Explorations, Farhat HACHED University Hospital, Sousse, Tunisia

**Keywords:** Cellular Reprogramming, Epigenetic, Genetic, Inflammaging, Longevity, Metaflammation

## Abstract

The expanding body of research that suggests aging may be controllable is examined in our literature review, drawing on insights from certain domains where physiological degeneration is potentially modifiable. The discovery of telomerase and its connection to cellular senescence, the epigenetic reprogramming of adult cells into pluripotent states, and the role of autophagy in longevity are encouraging scientific milestones, as they address key signaling pathways of aging including rapamycin, adenosine monophosphate-activated protein kinase, and forkhead box O transcription factors. Clinical innovations involving growth hormone, metformin, and dehydroepiandrosterone have shown demonstrable modifiable biological age’s markers, as evidenced by the thymus regeneration, immunorestoration, and insulin mitigation experiment. Furthermore, lifestyle-based tactics such as stress management, dietary optimization, exercise, and circadian alignment have become widely available resources for extending life expectancy. Sleep disturbance, poor nutrition, and psychological stress are key factors in the relationship between accelerated aging on one hand and persistent low-grade inflammation and metabolic dysfunction on the other hand (ie, inflammaging and metaflammation concepts). The advent of targeted therapies (eg, senotherapeutics and Sirtuin activators) and precision medicine tools (eg, polygenic risk scores and multi-cancer early detection tests) further highlight an ongoing shift from reactive to preventive medicine. While ethical and regulatory challenges—particularly regarding equitable access and long-term safety— are yet to be fully addressed, there is consensus that aging is a dynamic process open to intervention. This literature review urges researchers, physicians, and legislators to prioritize aging research, support translational initiatives, and integrate evidence-based treatments into public health frameworks.

## INTRODUCTION

Physicians with expertise in intensive care or sports medicine have witnessed firsthand the power of reversal of organ damage and therapeutic interventions [1, 2] from the healing of acute physiological disruptions due to drugs or muscle atrophy [1, 2], to how resistance training with a high-protein diet can modulate sarcopenia — a condition once thought to be irreversible, marking the point when aging becomes inevitable [3]. It is reasonable, then, to ask: Can we apply similar logic and modify or reverse aging? Indeed, recent scientific breakthroughs suggest that aging may be modifiable to some extent [4–6], fueling efforts to explore means of extending healthspan (ie, years lived free of diseases) and lifespan (ie, total years lived) [7, 8]. The urgency of this question is underscored by the growing health burden of aging-related diseases, which pose one of the most pressing healthcare challenges of our time. As the global healthspan-lifespan gap widens, healthcare systems face increasing strain from chronic diseases, long-term care, and disability services associated with population aging [9]. This economic reality, compounded by demographic projections that the global population aged 60 or older will double to two billion by 2050, creates an imperative for paradigm shifts in how we approach aging research and intervention [10]. The potential for aging interventions to reduce aging-related human suffering and economic burden makes a strong case for approaching aging (ie; reimagining aging), with the current tools, as a modifiable process.

Nowadays (ie, September 19, 2025), it is widely recognized that chronological age is different from biological age [11, 12]. While chronological age is the number of years a person has lived, biological age represents the vitality of – or how old– their cells and tissues are based on a multitude of aging biomarkers, including genetic and epigenetic changes [11–13]. Biochemical tests, telomere length assessment, and epigenetic clocks are used to estimate the individual’s biological age and, subsequently, the gap between biological and chronological ages. Changes in this gap can then be used to measure the effects of medicinal and other interventions aimed to delay or attenuate aging, with emerging interest in interventions that might potentially restore some aspects of youthful function [11, 12, 14]. From Nobel Prize-winning discoveries of telomere biology, which revealed how telomeres and the enzyme telomerase protect chromosomes and influence cellular aging, and the generation of pluripotent stem cells from fibroblasts [5, 16] to clinical trials showing thymic rejuvenation [6], the scientific foundation for modifying aging is only getting stronger. The scientific community stands at the precipice of a revolution, and it is long overdue for a call to action to bridge the gap between the current evidence [4, 17, 18] and the persistent, outdated notion that aging is an inevitable march toward decline [19]. Indeed, aging is a dynamic process ripe for intervention [4, 17, 18], and we hope that our present literature review will encourage the scientific community to invest in innovative therapies that can improve healthspan and lifespan, and bring them to clinic.

Our literature review aimed to narratively examine the emerging evidence suggesting that aging may be controllable rather than inevitable. Specifically, our objectives are to: (i) Narratively synthesize recent scientific breakthroughs that challenge traditional views of aging as an irreversible process; (ii) Highlight promising clinical applications of innovative discoveries that emerged from laboratory discoveries; and (iii) Advocate for a fundamental paradigm shift in how aging is conceptualized, researched, and treated in clinical settings. By organizing evidence from molecular mechanisms to clinical trials conducted recently, we provided a comprehensive framework for understanding aging as a potentially modifiable condition rather than an inexorable decline.

### Literature search and synthesis

To compile this narrative literature review, we conducted a literature search using PubMed/Medline, covering publications from its inception through May 7, 2025. The search strategy included the following keywords: Aging, Cellular Reprogramming, Epigenetics, Genetics, Healthspan, Lifespan, Inflammaging, Longevity, Metaflammation, and Telomeres. We prioritized original research articles, systematic reviews, and landmark papers relevant to recent scientific and clinical advances. In addition to the primary search, we examined the reference lists of key articles to identify additional pertinent publications. This approach ensured an up-to-date synthesis of the evolving landscape in aging research and its potential clinical implications.

### Insights from Nobel Prize-winning discoveries to cellular aging, rejuvenation, and longevity pathways

We learned from the Nobel Prize 2009 winning work in Physiology or Medicine [20] that the telomere’s length shortens with time and that telomerase is essential to rebuild the protective chromosomal ends [15]. Deficiency of this enzyme results in a gradual shortening of telomere repeats during successive cell divisions, limiting viability and leading to cell death in a process known as replicative senescence [20, 21]. Mutations in genes encoding components of the telomerase complex cause hereditary diseases predisposing to cancer and defects in stem cell renewal and tissue maintenance, to name a few [20]. The potential of telomerase activation in targeting deoxyribonucleic acid (DNA) methylation and multiple aging hallmarks, as demonstrated by the discovery of telomerase reverse transcriptase (TERT) activator compounds that can rejuvenate cells by reactivating telomerase [22]. However, this artificial activation is not without risks, since TERT promoter-activating mutations are common in many cancers [23–25]. Research that formed the basis for the Nobel Prize 2012 in Physiology or Medicine [26] demonstrated that cellular reprogramming is capable of turning mature, differentiated cells into pluripotent stem cells, erasing their developmental identity and epigenetic age. Research on partial cellular reprogramming is ongoing, with promising initial results suggesting that it may be possible to restore youthful function to aging cells [27]. These breakthroughs raised the tantalizing possibility of attenuating cellular aging [28]. If cellular rejuvenation is feasible, can we rejuvenate tissues or the human they form? In 2020, Sinclair’s work made ground-breaking contributions to aging research, with an experiment that reversed blindness in mice by reprogramming their retinal cells [5]. Although not directly tied to the 2016 Nobel Prize in Physiology or Medicine [29], subsequent research has deepened our understanding of autophagy—a cellular recycling process critical for maintaining health and longevity [30–32]. These advances have also highlighted that inhibition of the mechanistic target of rapamycin (mTOR) promotes autophagy, a protective mechanism involved in healthy aging [30, 31, 33]. Indeed, mTOR signaling is thought to contribute to aging by promoting cellular growth and proliferation, leading to cellular senescence and exhaustion [34]. Other undesired effects of mTOR include its role in metabolic disorders such as insulin resistance and obesity, as well as its overactivation in a variety of cancers, where contributing to their growth and progression [34]. Paradoxically, although rapamycin-mediated inhibition of mTORC1/S6K1 signaling is anticipated to confer beneficial effects, chronic treatment has been found to impair metabolic function [35, 36], leading to hyperlipidemia, reduced adipose tissue mass, glucose intolerance, insulin resistance, and ultimately a diabetes-like phenotype [37–39]. In preclinical models, prolonged administration of rapamycin has been associated with the development of glucose intolerance and insulin resistance, adverse effects that are reversible, yet clinically significant [39]. In humans, isolated reports, such as the discontinuation of long-term rapamycin therapy by ‘’Bryan Johnson’’ (Bryan Johnson’s Blueprint project [40]) due to recurrent infections, dyslipidemia, and hyperglycemia, further emphasize the potential clinical risks associated with its sustained use [41]. Besides anti-mTOR therapy (eg, Rapamycin and its derivatives such as everolimus and temsirolimus), other promising interventions to enhance healthspan and extend lifespan include targeting fork head box class O (FOXO) transcription factors [33, 42]. Mounting evidence supports the essential roles of FOXO proteins in the regulation of age-related diseases [43]. Notably, many FOXO stimulants (eg, Metformin, exercise, intermittent fasting, and nutrients like curcumin and Berberine) may modulate FOXO pathways, thereby contributing to substantial health benefits [42, 44–47].

### Emerging strategies to modulate human aging and promote longevity

In 2019, the thymus regeneration, immunorestoration, and insulin mitigation trial (TRIIM) led by Dr. Fahy provided compelling clinical evidence that aging-related change may be reversible in humans [6]. The study demonstrated that a combination of growth hormone, Metformin, and dehydroepiandrosterone could (i) Rejuvenate the thymus gland–a critical component of the immune system affected by aging–and (ii) Reduce biological age, as measured by epigenetic clocks [6]. While the trial has several limitations, including a small sample size of only nine participants, a lack of a control group, and preliminary results, it nonetheless represents a pioneering step in translating preclinical findings into clinical interventions. This success paved, the way for the ongoing TRIIM extension (TRIIM-X) trial, offering hope that targeted interventions may attenuate aging-related tissue and organ decline [48, 49]. Shortly after the original TRIIM study publication, scientists from Harvard published their paradigm-shifting book entitled “Lifespan: Why We Age and Why We Don’t Have to” [4]. In this book, the authors reveal the “Information theory of aging” and posit that aging is driven by the progressive loss of youthful epigenetic information; much like a scratched compact disc that can no longer play music correctly. The authors argued that reparation of this epigenetic information could restore youthful function to cells and tissues. They highlighted the role of Sirtuins (a family of histone deacetylases that play a key role in regulating key biological processes) in promoting longevity [4]. When stimulated, Sirtuins (i) Regulate energy metabolism, (ii) Maintain genome integrity by regulating DNA damage response and repair, (iii) Help cells cope with oxidative stress, and (iv) Modify aging, with some studies suggesting Sirtuins may promote longevity through epigenetic regulations [50, 51]. Sirtuins can be activated through various interventions, including supplementation with Nicotinamide adenine dinucleotide and Nicotinamide mononucleotide, exercise (especially high-intensity interval training), caloric restriction, and intermittent fasting [4]. Importantly, studies show that elderly individuals can safely engage in intensive intermittent exercise when a proper warmup is performed [52–55]. Conversely, stressors like poor sleep, excessive carbohydrate consumption, and advanced glycation end-products can inhibit Sirtuins, contributing to accelerated aging [56]. It is worth noting here that Resveratrol, a naturally occurring compound in some grapes and berries, is proposed to activate Sirtuins, although the evidence for its efficacy remains inconclusive and not universally accepted [57]. Resveratrol, once hailed as a longevity molecule, exemplifies the pitfalls of premature hype [4]. Early studies linking Resveratrol to improved lifespan in yeast were later questioned due to experimental artifacts [4, 58]. Despite mixed clinical outcomes, Resveratrol continues to be widely marketed, highlighting the supplement industry’s conflict-ridden landscape [59]. A lesson repeatedly learned is that rigorous trials are essential to (i) Separate hope from hype [60] and (ii) Ensure that positive signals in basic research translate into clinically meaningful outcomes before marketing. This is critical because, while many anti-aging supplements showed promise in extending lifespan and enhancing healthspan in animals (T0 phase of translational research), their efficacy and long-term safety in humans remained unproven, with overall weak evidence to support widespread use [61]. In contrast, addressing nutritional deficiencies that directly contribute to aging offers a more grounded approach. For instance, Magnesium has been shown to influence multiple hallmarks of aging [62, 63] as defined by Lopez-Otin et al. [17]. Therefore, in the midst of continuous efforts to investigate and validate more complex anti-aging medicines, preventing or correcting such inadequacies is a straightforward and practical method in supporting healthy aging and should not be underestimated.

Another interesting molecular pathway under investigation is adenosine monophosphate-activated protein kinase (AMPK), which is being targeted with senotherapeutics [64–66]. AMPK controls cellular homeostasis, metabolism, resistance to stress, cell survival and growth, cell death, and autophagy–all are critical determinants of aging [64]. Senotherapeutics are an emerging class of drugs designed to target the biological drivers of aging by either eliminating senescent cells (senolytics) or suppressing the senescence-associated secretory phenotype (senomorphics) [65, 66].

### Inflammaging, metaflammation, and the metabolic roots of aging

A critical aspect of the aging process is inflammaging, which is the chronic, low-grade inflammation that develops with age and is recognized as both a contributor to the underlying mechanisms of biological aging and an accelerator of its progression [17, 67–69]. Inflammaging results from the intricate interaction of senescent cells, compromised autophagy, mitochondrial dysfunction, and dysregulated immune responses [17, 67–70]. This inflammatory state is marked by increased circulating cytokines has a measurable impact on biological age as measured by indicators like the Horvath epigenetic clock, and contributes to aging-related health conditions, such as cardiovascular disease, dementia, and frailty syndrome [8, 71]. While inflammaging underscores the role of chronic inflammation in aging, emergent research reveals that metabolic dysfunction can accelerate this process long before traditional aging markers appear. This phenomenon, termed metaflammation [72], links modern lifestyle epidemics- such as obesity and insulin resistance- to premature aging [73, 74]. Unlike inflammaging, which unfolds over decades, metaflammation is a modifiable driver of aging, fueled by nutrient excess, processed food-based diets, adipose tissue dysfunction, and mitochondrial stress. It hijacks the same inflammatory pathways (eg, nuclear factor kappa-light-chain-enhancer of activated B cells, NODlike receptor family, pyrin domain containing 3, mTOR, senescence) [75–78] as inflammaging, but does so earlier and more aggressively, leading to accelerated cellular senescence and epigenetic aging [79]. The interplay between metabolic health and inflammation suggests that addressing metaflammation through lifestyle modifications could effectively promote healthy aging and help mitigate the long-term effects of inflammaging. This link positions metabolic health as a powerful lever to delay the onset of inflammaging and exemplifies the paradigm shift toward preventive approaches in aging interventions. In addition to its effect on inflammaging and metainflammation, chronic stress is a significant contributor to inflammation by triggering a cascade of physiological responses that influence both mental and physical well-being. When the body perceives stress, the hypothalamic-pituitary-adrenal axis is activated, leading to the release of stress hormones like cortisol [80]. While this response is adaptive in the short-term, prolonged exposure to these hormones disrupts homeostasis, promoting systemic inflammation, immune dysfunction, oxidative stress, and gut-brain axis dysbiosis [81]. These changes not only increase the risk of anxiety, depression, and metabolic disorders, but also perpetuate both inflammaging and metaflammation. Thankfully, there are natural ways to successfully combat these inflammatory mediators. A 2024 meta-analysis demonstrated that physical activity significantly reduces depressive symptoms with effect sizes comparable to those of conventional therapies [82]. Additionally, a 2025 narrative review highlighted that regular aerobic and resistance training is beneficial for older adults in mitigating their cognitive decline and enhancing their overall well-being [83].

The above facts, findings, and investigated pathways related to the science of aging are not without precedent [84]. Sarcopenia, a condition characterized by age-related muscle loss [3], was officially listed as a disease in 2016 under the International Classification of Diseases, 10^th^ Revision, Clinical Modification [85]. Importantly, sarcopenia is both treatable and reversible through interventions like adequate protein intake and resistance training [86–88]. If sarcopenia, a hallmark of aging, can be reversed, it raises the question: why not aging itself?

However, while extending lifespan and healthspan is crucial, it is equally important to live a life with purpose, joy, and fulfillment [89]. Individuals should not feel compelled to pursue extreme longevity measures at the expense of quality of life [89]. For instance, Bryan Johnson’s commitment to Project Blueprint’s team [40] is admirable, yet the heavy investment and unrealistically extreme health measures employed highlight the need for balance. We suggest that physicians, as they treat their patients, focus on finding harmony between longevity and living life to the fullest, enjoying relationships, experiences, and personal growth.

### The sleep-aging connection: How sleep quality, melatonin, and circadian health shape the aging process

A growing body of research highlights the bidirectional relationship between aging and sleep. On one hand, sleep quality and duration often diminish as we age [90–92], frequently influenced by chronic medical and psychiatric illnesses, increased stress, and the use of multiple medications [90–92]. On the other hand, poor sleep, especially in older adults, is associated with a higher risk of cognitive decline, falls, and death [93, 94].

Mechanistic studies have shown that sleep disturbances can accelerate several hallmarks of aging, including genomic instability, mitochondrial dysfunction, and cellular senescence [95–98]. Research has elucidated the molecular mechanisms linking circadian disruption to cellular senescence through multiple interconnected pathways [99–101]. Core circadian clock genes, including CLOCK, BMAL1, PER, and CRY, form the foundation of the molecular clockwork that regulates cellular metabolism and stress responses, thereby influencing senescence pathways [99]. These genes operate through negative feedback loops where CLOCK-BMAL1 heterodimers upregulate target genes, including Period and Cryptochrome, whose protein products then inhibit CLOCK-BMAL1 activity, maintaining circadian rhythm homeostasis [99]. Disruption of these core clock components leads to metabolic dysfunction, increased oxidative stress, and accelerated cellular aging through dysregulation of clockcontrolled genes that govern fundamental cellular processes. There is an established bidirectional relationship between aging and circadian function: aging leads to significant changes in circadian rhythms, including dampened amplitude and phase advances in sleep-wake cycles, while disrupted circadian timing itself accelerates aging processes [99]. Additionally, the extracellular matrix environment can regulate circadian clocks in a cell-type-dependent manner, demonstrating how tissue structure changes throughout life impact the molecular control of circadian cycles. The senescence-associated secretory phenotype is also subject to circadian regulation, with inflammatory cytokine secretion following diurnal patterns that can disrupt tissue-level coordination [100, 101].

Melatonin, a hormone critical for regulating circadian rhythms and sleep, declines significantly with age. This phenomenon has been implicated in both the increased prevalence of sleep disorders and the pathogenesis of age-related neurodegenerative diseases [95, 102, 103]. Clinical and preclinical studies suggest that melatonin supplementation may not only improve sleep quality but also exert antioxidant, anti-inflammatory, and neuroprotective effects, potentially slowing cognitive decline and protecting against conditions like Alzheimer’s disease [104–107]. Evidence also suggests that melatonin supplementation may confer protective cardiovascular effects via blood pressure regulation, improved endothelial function, and attenuation of atherosclerosis [103, 108, 109]. These findings emphasize the importance of maintaining sleep health and circadian rhythm integrity as part of the strategy aimed at promoting healthy aging. While aging is often framed as an inexorable decline in physiological functions, marked by a reduction in melatonin production and associated sleep disruptions [110], emerging evidence argues that we have significant power to intervene. Modern lifestyle factors, such as exposure to artificial light at night [111] and electromagnetic fields from electronic devices [112] disrupt circadian biology and further impair sleep quality. Additionally, a diet rich in processed foods impacts the gut microbiome by reducing the intake of sleepsupporting fibers [113] and increasing metaflammation, which can further disrupt sleep architecture [114]. Even fluoride exposure from toothpaste may impair pineal gland function and subsequently sleep regulation [115–117]. Luckily, a variety of preventative measures can be employed to maintain and even restore healthy sleep patterns. These measures include (i) Morning sunlight exposure, which helps reset circadian clocks by activating intrinsically photosensitive retinal ganglion cells [118], (ii) Consuming tryptophan-rich whole foods to support melatonin synthesis [119, 120], (iii) Grounding practices to normalize cortisol rhythms [121], and (iv) Body temperature modulation by keeping the bedroom cool [122]. When these interventions combined with regular exercise [123], reduced evening screen time [124, 125], and earlier meal timing [126, 127], they can significantly mitigate the risk of sleep disturbances commonly associated with aging. The populations in Blue Zone areas known for exceptional longevity and health [128] where lifestyle optimization supports robust sleep quality well into advanced age exemplify this holistic approach.

### From irreversible decline to modifiable process: The evolving paradigm of aging and its clinical, ethical, and societal implications

It is worth noting that the conceptual view of aging has evolved significantly over time from the outdated passive, irreversible process to a dynamic, modifiable phenomenon [Box 1]. This paradigm shift has profound clinical implications. Whereas traditional approaches focused primarily on treating the symptoms of aging, modern strategies increasingly target the drivers of the process–such as epigenetic changes and dysregulation of molecular signaling pathways [4, 17–19]. The new approach, grounded by advances in geroscience, epigenetics, and medical innovations, emphasizes the potential for slowing or mitigating aspects of aging [17, 18]. While this approach offers promising opportunities for anti-aging interventions, it also introduces complex ethical, societal, and regulatory challenges [129]. Key concerns include long-term safety, unintended consequences of modifying aging processes, and the need for robust regulatory oversight [130]. In addition, we are already witnessing privileged access to longevity measures by affluent populations, which would potentially further widen health disparities and create a biologically privileged class. In addition, extending lifespans may have significant societal implications, such as straining healthcare systems, altering workforce dynamics, and affecting intergenerational equity. However, concerns about health and socioeconomic disparities may have been exaggerated. The Blue Zone communities showed us that low-cost lifestyle interventions can help people live long, healthy lives [128], rather than relying solely on high-income status or expensive medical care—an approach often associated with high-gross domestic product nations [131]. This is further supported by the fact that, the United States, despite its high gross domestic product, exhibits the world’s largest lifespan-healthspan gap [9], underscoring that wealth alone does not guarantee healthy longevity.

Box 1 Aging perspectives evolution over time

**Table:** 

	Old perspective of aging	New perspective of aging
**Philosophical foundation**	Inevitable Linear process Passive acceptance	Dynamic Modifiable process Active intervention

**Mechanistic focus and biological basis**	Wear and tear Linear progression governed by immutable biological laws (eg; mitochondria dysfunction: FRMTA, DNA mutations, immunologic theory) Irreversible damage	Mitochondria dysfunction: mtDNA mutations Loss of epigenetic information (eg; DNA methylation, Sirtuins activation, Telomere attrition) Dysregulated molecular signaling pathways (eg; mTOR, FoxO, AMPK) “Reversible” modifications

**Clinical approach**	Managing age-related diseases Reactive	Targeting aging as a root cause Proactive

**Key outcomes**	Manage decline Static lifespan and healthspan	Preventive approaches Potential to extend healthspan and lifespan

**Societal impact**	Unavoidable burden Limited discourse Passive acceptance	Transformative ethical questions Healthspan extension Active intervention

**Ethical priorities**	End of life care	Equity Safety and commercialization risks

AMPK: Adenosine monophosphate-activated protein kinase. DNA: Deoxyribonucleic acid. FoxO: Fork head box class O. FRMTA: Free radical mitochondrial theory of aging. mtDNA: Mitochondrial DNA. mTOR: Mechanistic target of rapamycin.

Addressing these issues will require interdisciplinary collaboration, transparent public dialogue, and careful evaluation of both the benefits and risks as the field advances [129]. One notable development in this regard is the growing focus on healthy longevity medicine [132], which shifts the goal from increasing lifespan to enhancing healthspan, increasing the years spent living without disability and chronic disease [133, 134]. This entails closing the lifespan-healthspan gap [135] by prioritizing primary prevention over treating symptoms. Despite a three-decade improvement in life expectancy since the mid-twentieth century, healthspan has not kept pace, largely due to the global burden of non-communicable diseases in aging populations [9]. Without meaningful action to improve healthspan, this gap is poised to widen. Demographic projections anticipate that by 2050, the number of people over 60 will double to two billion, those over 80 will triple. By 2075, nearly 60 people aged 65 or older will depend on 100 working-age individuals, doubling today’s ratio [10]. Investing in healthspan can mitigate these socioeconomic challenges and could even yield a positive outcome by fostering a more independent and functional older population [133].

### Conditional transition from current unsatisfactory present to future resilience: Science-backed and technology-driven paths to a healthier aging

Despite alarming trends–including (i) 22% of global mortality being related to poor dietary habits [136], (ii) Projected 77% and 90% increase in global cancer cases and deaths by2050, respectively [137], (iii) 60% of Americans live with at least one chronic illness and 40% with two conditions [138], and (iv) A widening global lifespan-healthspan gap that reached 9.6 years (12.4 years in the US) [9] –there are numerous actionable scientific advancements [Table 1] that could dramatically alter these outcomes if effectively implemented [61].

**Table 1:** Emerging anti-aging interventions

Intervention	Mechanism of action	Evidence level	Limitations / risks
**Telomerase activators**	Elongate telomeres, maintain chromosomal stability	Preclinical, early pilot human	Oncogenic potential due to TERT reactivation

**Partial cellular reprogramming**	Epigenetic reset, restoration of youthful gene expression	Preclinical (animal models)	Tumorigenesis; no human trials yet

**Rapamycin & mTOR inhibitors**	Inhibit mTOR → promote autophagy, reduce senescence	Strong preclinical, limited pilot human	Insulin resistance, dyslipidemia, infections

**Metformin**	AMPK activation, improves insulin sensitivity, reduces oxidative stress	Observational & randomized clinical trials	GIT side effects; unclear longevity effect in healthy humans

**Sirtuin activators (NAD^+^, NMN)**	Maintain genome integrity, regulate energy metabolism	Preclinical & early human pilot	Long-term safety unknown

**Resveratrol**	Proposed SIRT1 activation, antioxidant effects	Mixed preclinical, weak/ inconsistent human	Clinical efficacy unproven

**Lifestyle interventions (exercise, diet, sleep, stress)**	Multi-target modulation of hallmarks of aging	Strong human evidence	Adherence variability

**Magnesium supplementation**	Cofactor in DNA repair, mitochondrial function, oxidative stress reduction	Clinical & epidemiological studies	Benefits strongest in deficient individuals

**Melatonin (Elderly supplementation)**	Antioxidant, anti-inflammatory, and neuroprotective effects, circadian rhythms and sleep regulations	Clinical & preclinical studies	Sedative effect with increased falls risk, drug interference with increased hemorrhagic risk if Warfarin prescription

Notes: Evidence levels classified as preclinical, pilot human, clinical, or systematic.

AMPK: Adenosine monophosphate-activated protein kinase. DNA: Deoxyribonucleic acid. GIT: Gastrointestinal tract. NAD^+^: nicotinamide adenine dinucleotide. NMN: Nicotinamide mononucleotide. mTOR: Mechanistic target of rapamycin. SIRT1: Sirtuin isotype1. TERT: Telomerase reverse transcriptase.

For instance, Topol [61] highlighted in his latest book that individuals have significant agency to change their lifestyle to improve their health by adopting a more balanced diet, quality sleep, regular exercise, a healthy environment, and strong social connections. Advances in artificial intelligence, OMICS, personalized medicine, and precision medicine [139] support this comprehensive lifestyle approach. According to Topol [139], these technologies are converging to revolutionize our understanding of aging and age-related diseases. To further demonstrate this, polygenic risk scores can be used to identify people who are at high risk for specific diseases, allowing for early intervention and prevention [140]. Multi-cancer early detection tests also have great potential to revolutionize early cancer detection through spotting circulating cell-free DNA from a simple blood sample, urine, saliva, or other body fluids, with arguably accurate localization of multiple cancer types at very low and fixed false-positive rates [141]. Furthermore, artificial intelligence-powered genomics and epigenomics are now able to predict genome instability and link nuclear morphology with clinically relevant prognostic biomarkers across multiple cancer types [142]. We can anticipate significant increases in human lifespan and health as these technologies develop, so long as evidence-based practices are properly applied.

### Strengths and limitations

There are certain strengths to our thorough narrative synthesis. First, it skillfully combines a wide variety of contemporary scientific discoveries, such as Sirtuins, cellular reprogramming, autophagy, telomere biology, senotherapeutics, and the metabolic foundations of aging (eg; AMPK and mTOR). In order to integrate various but related processes in aging biology, such a narrative approach is essential. Second, our paper examines clinical therapies that offer practical, early clinical evidence for modulating biological markers of aging, such as the TRIIM trial, although it is small, uncontrolled, and the results remain preliminary [6] and its extension TRIIM-X [48, 49]. This emphasis on translation is in line with the expanding field of geroscience, where it is essential for establishing a connection between patient outcomes and molecular biology [143]. Third, beyond biological processes, our paper also stresses lifestyle (ie; exercise, sleep, nutrition), highlighting changeable environmental influences. This holistic perspective mirrors findings from the Blue Zones research [128] and underscores that aging therapies are not primarily pharmaceutical. Fourth, our paper discusses ethical concerns such health inequities, access to medicines, and the effects of lifetime extension on society (eg, economic implications, equity). This is in line with more general conversations in the field [144]. Lastly, references to polygenic risk scores, multi-cancer early detection tests, and epigenetic clocks [71] demonstrate a current understanding of precision medicine techniques.

There are certain restrictions on this literature review as well. First, our paper lacks the formal rigor of a systematic review, even though we have synthesized a large body of material. By describing search tactics, inclusion criteria, and bias evaluation, systematic reviews increase the dependability of findings [145]. Second, although our paper joyfully presents encouraging results, it does not always address the drawbacks or disputes of these approaches. For instance, the TRIIM study’s small sample size (n=9) raises doubts about generalizability [6], and the evidence supporting resveratrol in humans is still equivocal [57]. Our “moderate” tone concerning aging modulation fully fit with Topol’s [61] emphasis on distinguishing between hype and hope due to this omission. Third, our narrative assessment has the risk of cherry-picking data, even though it is useful for broad insights [146]. The emphasis on “full reversibility,” for example, can obscure the more widely accepted view that aging is not entirely reversible but only partially so [17]. Fourth, while we mentioned ethical and regulatory challenges, we do not delve into the crucial issue of long-term safety and potential unintended consequences (eg, cancer risks with telomerase activation, or metabolic trade-offs of rapamycin) [147]. Finally, anti-aging interventions do not exert uniformly positive effects but instead operate through complex and occasionally paradoxical mechanisms that demand nuanced interpretation and context-specific evaluation [148]. For instance, the exacerbation of insulin resistance and impaired glucose tolerance observed with rapamycin administration has been attributed by some studies to its “starvation-mimetic” properties rather than to an inherently diabetogenic action [148]. Notably, these metabolic perturbations appear to be separable from the drug’s longevity-promoting benefits, indicating that a single intervention may concurrently enhance lifespan while eliciting adverse metabolic consequences [148]. In brief, our paper provided an impressive, comprehensive, and upto-date narrative that captures the optimism and momentum in aging research. However, it does not provide formal systematic review rigor or critical evaluation of potential risks and controversies.

## CONCLUSIONS

Aging should not be seen as an inevitable and passive fate [4, 17], but instead a dynamic and modifiable process. Science has entered a new era marked by breakthroughs in telomere maintenance, epigenetic reprogramming, autophagy enhancement, and lifestyle adjustments [4, 5, 15, 17, 30, 31, 34] – all of which have been shown to decelerate or even partially reverse biological aging [4, 128]. However, some interventions can carry significant risks, such as telomerase reactivation that may promote cancers [23–25], and the prolonged use of rapamycin that may lead to serious metabolic derangements [39]. To accelerate progress in clinically meaningful aging-modifying interventions, collaboration among governments, academic institutions, and private companies is essential. The scientific community should prioritize translational research and precision medicine, ensuring that healthspan receives equal, if not greater, attention compared to lifespan extension [131, 134, 149–151]. Policymakers need to (i) Treat aging as a modifiable condition in their research funding priorities, (ii) Integrate geroscience into chronic disease management guidelines, (iii) Train healthcare professionals in longevity medicine, and (iv) Build national registries to monitor long-term outcomes of aging interventions. Finally, public education and empowerment are crucial. This includes adopting evidence-based strategies such as regular exercise, healthy sleep, intermittent fasting, stress management, cognitive stimulation, and social connections, and consuming science-backed dietary supplements.

### The take-home message

– Aging is increasingly being recognized as modifiable phenomenon.
– Recent advances in science, including cellular reprogramming, telomerase activation, lifestyle changes, and treatments for metabolic dysfunction and inflammation, aid in realizing the hope of modifying aging.
– Translational research is crucial to bridge the gap between these discoveries and clinical practice, but must be conducted with ethical, societal, and regulatory considerations to ensure equitable access and long-term safety in mind.
– A paradigm shift is underway: aging is becoming a treatable condition, opening the door for healthier, longer lives.

## Data Availability

Data sharing not applicable to this article as no datasets were generated or analyzed during the current study.

## Declaration

The authors wish to disclose that two artificial intelligence tools (ie, ChatGPT ephemeral, QuillBot) were utilized to enhance the manuscript’s wording, readability, and language quality. The tools were used only for language refinement and not for generating text [152, 153].
